# Metformin enhanced the effect of pirfenidone on pulmonary fibrosis in mice

**DOI:** 10.1111/crj.13731

**Published:** 2024-01-18

**Authors:** Nana Liu, Yanqiu Song, Ting Liu, Hongyu Wang, Naihao Yu, Hui Ma

**Affiliations:** ^1^ Department of Critical Care Medicine Tianjin Academy of Traditional Chinese Medicine Affiliated Hospital Tianjin China; ^2^ Cardiovascular Institute Chest Hospital, Tianjin University Tianjin China; ^3^ Department of Respiratory and Critical Care Medicine Chest Hospital, Tianjin University Tianjin China

**Keywords:** bleomycin, idiopathic pulmonary fibrosis, metformin, pirfenidone, ROS

## Abstract

**Background:**

The aim of the study is to observe the anti‐inflammatory and antioxidative stress effects of metformin on bleomycin (BLM)‐induced pulmonary fibrosis in mice.

**Methods:**

Mice with BLM‐induced pulmonary fibrosis were treated with pirfenidone, metformin, pirfenidone plus metformin and the NADPH oxidase 4 (NOX4) inhibitor diphenyleneiodonium chloride (DPI). Pathological changes and hydroxyproline (HPO) levels were examined in the lung tissue of mice with pulmonary fibrosis. Superoxide dismutase (SOD) activity and malonaldehyde (MDA) levels in lung tissue were determined.

**Results:**

Compared with pirfenidone, pirfenidone plus metformin could reduce alveolar damage and collagen fibre deposition and alleviate BLM‐induced pulmonary fibrosis. Lung HPO levels were significantly lower in the PFD + MET group than in the BLM group (*p* < 0.05). SOD levels in the lungs of mice were increased in the PFD + MET group than in the BLM group (*p* < 0.05). Metformin and pirfenidone plus metformin can reduce MDA levels (*p* < 0.05). Pirfenidone plus metformin could reduce HPO levels, increase SOD levels, and reduce MDA levels in the lungs of mice. There was a significant correlation between the HPO level and the Ashcroft score (r = 0.520, *p* < 0.001).

**Conclusion:**

Metformin enhanced the antifibrotic effects of pirfenidone on BLM‐treated mice. Moreover, these findings provide an experimental basis for examining whether metformin can improve the antifibrotic effects of pirfenidone on patients with idiopathic pulmonary fibrosis (IPF). It has broad therapeutic prospects for patients with IPF.

## Graphical abstract

Metformin enhanced the antifibrotic effects of pirfenidone on bleomycin‐induced mice. The antioxidant stress effect of metformin may be related to the inhibition of NOX4. Moreover, these findings provide an experimental basis examining whether metformin can improve the antifibrotic effects of pirfenidone on patients with idiopathic pulmonary fibrosis. It has broad therapeutic prospects in patients with IPF.

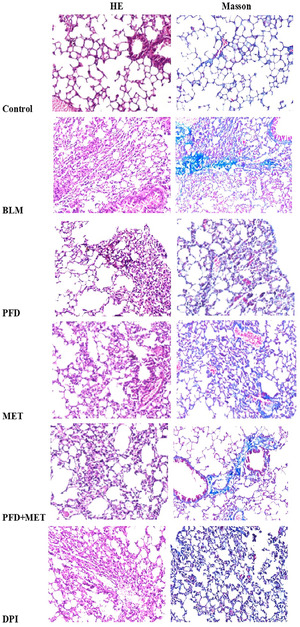

## 1 INTRODUCTION

Idiopathic interstitial pneumonia (IPF) is a specific variety of chronic progressive pulmonary fibrosis. The breakdown of alveolar structure and aberrant extracellular matrix deposition in the lung interstitium are its defining characteristics. The onset of the disease is insidious, many complications occur when patients are diagnosed, and the prognosis is poor. The clinical manifestations are dyspnoea and dry cough, which gradually worsen. The deterioration of lung function is irreversible. Most patients will delay seeing a doctor, and the median survival after diagnosis is only 2–3 years.^1^ Current evidence suggests that oxidative/antioxidant imbalance plays an important role in the development of pulmonary fibrosis. Oxidative stress can cause epithelial cell necrosis, upregulate the expression of fibrotic cytokines, and cause an imbalance in lung cathepsin and antiprotease. This eventually leads to the accumulation of large amounts of extracellular matrix and the development of pulmonary fibrosis. Pirfenidone has been used in the clinical treatment of IPF. However, there are some reactions and negative gastrointestinal effects, and its curative effect is limited. Metformin is the most commonly used hypoglycaemic drug. In addition to hypoglycaemic effects, it also reduces inflammation and antioxidant stress.^2^, ^3^ This study investigated the anti‐inflammatory and antioxidant stress effects of metformin on bleomycin (BLM)‐induced pulmonary fibrosis in mice.

## 2 METHODS

### 2.1 Mouse model

Forty‐eight male mice (C57BL/6J; 6–8 weeks old, weighing 21–25 g, SPF level) were purchased from Beijing Vital River Laboratory Animal Technology Co., Ltd. (Beijing, China). The mice were fed under the same conditions in an optimal living environment.

### 2.2 Establishment of a pulmonary fibrosis animal model and drug intervention

Forty‐eight mice were randomly divided into six groups with eight mice in each group: the control group, BLM group, pirfenidone (PFD) group, metformin (MET) group, pirfenidone+MET group (PFD + MET), and NADPH oxidase 4 (NOX4) inhibitor diphenyleneiodonium chloride (DPI) group. Every group except the control group was given 1.5 U BLM (1.5 mg/kg; Nippon Kayaku Co., Tokyo, Japan) by endotracheal administration on Day 0. Beginning on Day 7, the PFD group was intragastrically administered pirfenidone (200 mg/kg body weight; Beijing Kangdini Pharmaceutical Co., Ltd., China) once daily. The MET group was intragastrically administered metformin (300 mg/kg body weight; Selleck Biotechnology Co., Ltd., USA) once daily.^4^ Pirfenidone and metformin were administered to the PFD + MET group. The DPI group was injected with DPI (1 mg/kg body weight; Santa Cruz Biotechnology Co., Ltd., USA) once daily. The control group and the BLM group were given the same volume of saline every day. The mice were sacrificed on Day 21, and the lungs were collected for histological staining, hydroxyproline (HPO) assays, and oxidation–reduction analysis.

### 2.3 Histological analysis

Left lung tissue was immersed in paraffin wax three times for 45 min each. The paraffin‐embedded tissues were cut into 5 μm sections by a microtome and then were subjected to haematoxylin‐eosin (H&E) and Masson's trichrome staining. The degree of inflammation and fibrosis in the lung tissue of mice was observed under an optical microscope. The Ashcroft score was used to categorize the severity of pulmonary fibrosis with H&E staining as previously described.^5^ Two pathologists made blinded evaluations. Ten visual fields were randomly selected from each sample, and the average score was the fibrosis degree score. The scoring was consistent between the two pathologists.

### 2.4 HPO assay

HPO levels in lung tissue was determined by alkaline hydrolysis. A microassay kit (Nanjing Jiancheng Bioengineering Institute, Nanjing, China) was used according to the instructions. The lung tissue (30–100 mg) was placed into the test tube, 1 mL of hydrolytic solution was added, and the sample was mixed well. After being covered, the samples were hydrolyzed at 95°C or in a boiling water bath for 20 min (mixed once after 10 min). The pH value of the sample was approximately 6.0–6.8, and then, double steaming water was added to 10 mL and mixed well. Three to four millilitres of diluted hydrolysate was added to an appropriate amount of activated carbon (~20–30 mg) and mixed well. The sample was centrifuged at 3500 RPM for 10 min, and 1 mL of supernatant was carefully used for detection.

The calculation formula is as follows:

$$\begin{array}{l} {\text{Hydroxyproline}\;\text{level}\;\text{in}\;\text{the}}_{\text{right}\;\text{lung}}\left(\mathrm{\mu g}/\mathrm{mg}\;\text{tissue}\right)\\ \;=\left(A_{\text{determination}}-A_{\text{blank}}\right)/\left(A_{\text{standard}}-A_{\text{blank}}\right)*C_{\text{standard}}\\ {}*V_{\text{hydrolysate}}/W\left(\text{tissue}\;\text{mass}\right). \end{array}$$

### 2.5 Superoxide dismutase (SOD) and malonaldehyde (MDA) level determination

SOD and MDA levels in lung tissues were determined with a microassay kit (Nanjing Jiancheng Bioengineering Institute, Nanjing, China) according to the instructions. The lung tissue was homogenized with normal saline (g:mL = 1:9). The samples were centrifuged at 3000 rpm for 10 min, and the supernatant was collected.

### 2.6 Statistical analysis

Statistical analyses were performed using GraphPad Prism 7 (GraphPad Software, La Jolla, California, USA). The data are reported as the mean ± standard error of the mean (SEM). The comparison of the difference among the single factor and multiple groups was performed using one‐way ANOVA and post hoc analysis with Tukey's multiple comparison. The differences among the two factors and multiple groups were analysed using two‐way ANOVA, and Tukey's multiple analysis was used to compare the differences between groups. Correlation analyses between Ashcroft score and HPO in lung tissue were with Spearman rank correlation analysis. *p‐*Values <0.05 were considered to indicate statistical significance.

## 3 RESULTS

### 3.1 Pirfenidone plus metformin protects against BLM‐induced pulmonary fibrosis in mice

**FIGURE 1:**
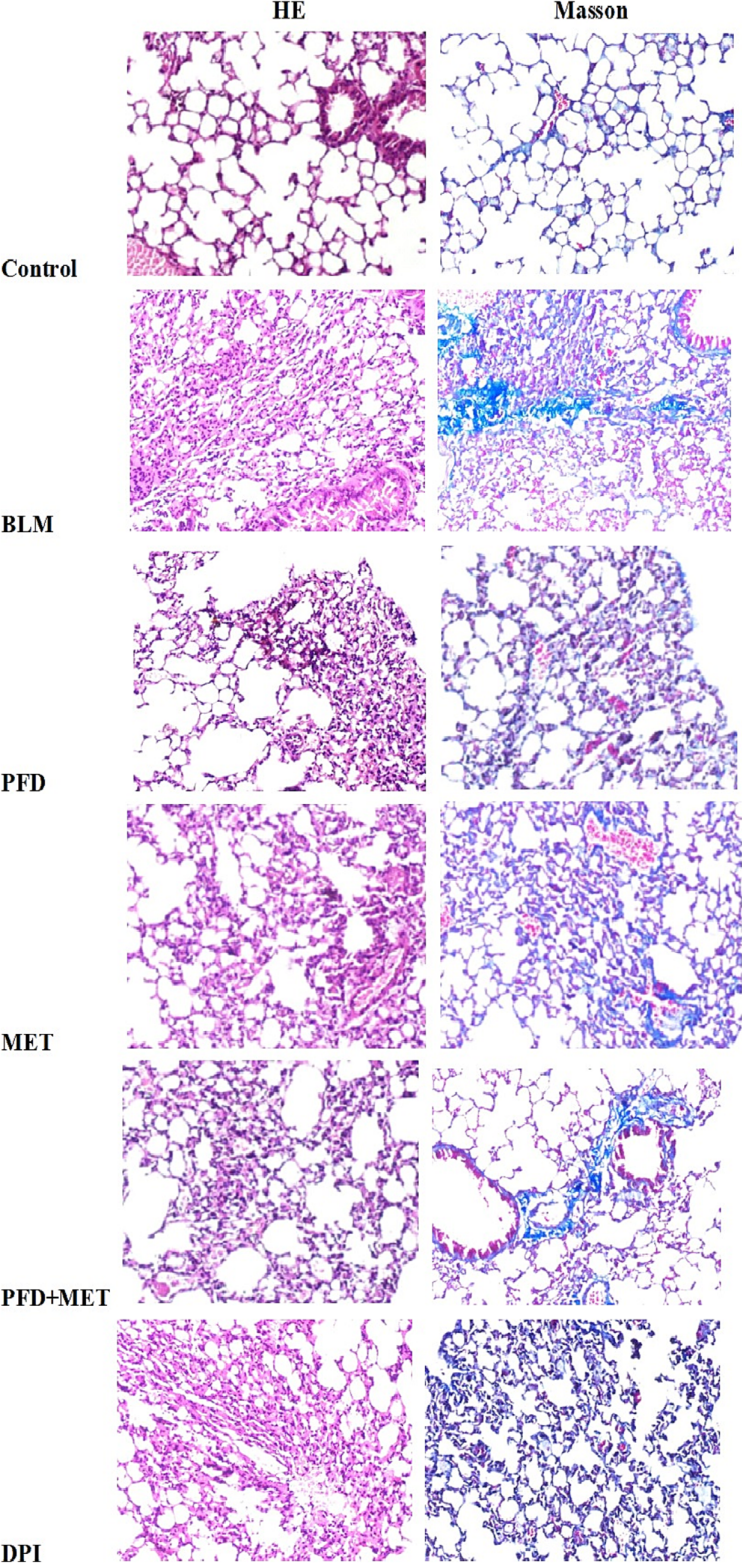
Pirfenidone plus metformin protects against bleomycin‐induced pulmonary fibrosis in mice. H&E staining revealed structural disruption in the lung tissue of mice given intratracheal bleomycin. A cytological microscope with a 20 × objective lens was used for analysis. The alveolar septa were significantly thickened and filled with fibrous tissue and extracellular matrix. The deposition of collagen fibres was increased in bleomycin‐induced lung injury, as shown by Masson's trichrome staining. Compared to pirfenidone alone, pirfenidone plus metformin could minimize lung fibrosis in mice.

**FIGURE 2:**
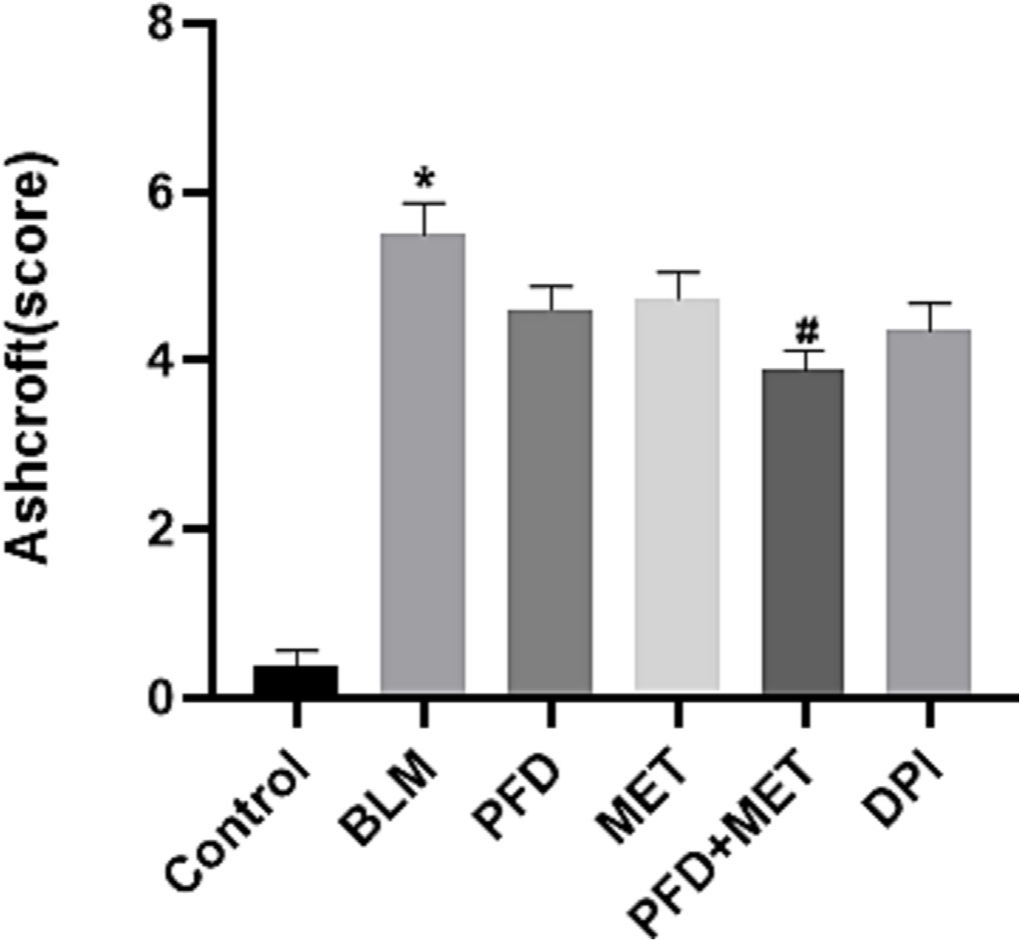
Ashcroft scores. Compared to pirfenidone alone, pirfenidone plus metformin could minimize lung fibrosis in mice. Compared with the control group, *p < 0.05. Compared with the bleomycin (BLM) group, #*p* < 0.05. Statistical analyses were performed by one‐way ANOVA with Tukey's test for comparisons between groups.

On Day 21, H&E staining revealed structural disruption in the lung tissue of mice that were administered intratracheal BLM. The alveolar septa were significantly thickened and filled with fibrous tissue and extracellular matrix (Figure 1). The deposition of collagen fibres was increased in BLM‐induced lung injury, as shown by Masson's trichrome staining (Figure 1). Decreased alveolar damage and collagen fibre deposition were observed in the mouse lung sections after the administration of pirfenidone, MET, pirfenidone plus MET, or DPI (Figure 1). The Ashcroft scores in the six groups (control, BLM, PFD, MET, PFD + MET, and DPI) were 0.375 ± 0.183, 5.500 ± 0.378, 4.625 ± 0.263, 4.750 ± 0.313, 3.875 ± 0.227, and 4.375 ± 0.324 (mean ± SEM), respectively. Compared with the BLM group, the PFD + MET group showed alleviated BLM‐induced pulmonary fibrosis (*p* < 0.05). Compared to pirfenidone alone, pirfenidone plus metformin could minimize lung fibrosis in mice (Figure 2).

### 3.2 Pirfenidone plus metformin decreased HPO levels in the lung tissue of BLM‐induced mice and the HPO level was correlated with the Ashcroft score

**FIGURE 3:**
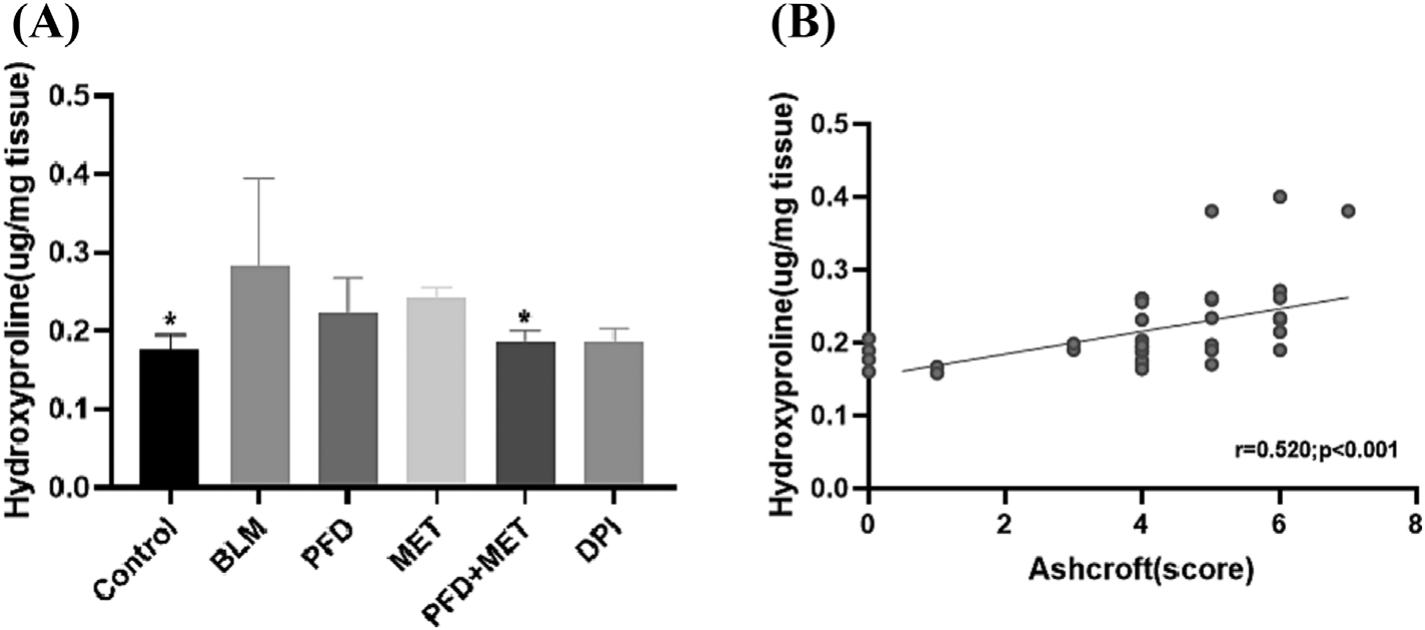
Pirfenidone plus metformin decreased hydroxyproline (HPO) levels in the lung tissue of bleomycin‐induced mice, and HPO levels were correlated with Ashcroft scores (A) HPO is a unique amino acid in collagen. It plays a crucial role in the synthesis of collagen and serves as a biochemical marker of pulmonary fibrosis. Compared with the bleomycin (BLM) group, *p < 0.05. Statistical analyses were performed by one‐way ANOVA with Tukey's test for comparisons between groups. (B) Scatter plot of the correlation between the Ashcroft score and HPO level in lung tissue. There was a positive correlation between the Ashcroft score and HPO level in the lung tissue of the six groups of mice (r = 0.520, *p* < 0.001). Correlation analyses were performed using spearman rank correlation analysis.

On Day 21, the HPO levels in the six groups (control, BLM, PFD, MET, PFD + MET, and DPI) were 0.176 ± 0.008, 0.247 ± 0.034, 0.222 ± 0.027, 0.242 ± 0.013, 0.193 ± 0.006, and 0.182 ± 0.008 μg/mg tissue (mean±SEM), respectively. Lung HPO levels were significantly lower in the PFD, MET, PFD + MET, and DPI groups than in the BLM group (Figure 3A). There was a significant correlation between the HPO level and Ashcroft score (r = 0.520, *p* < 0.001, Figure 3B).

### 3.3 Pirfenidone plus metformin reduces the level of oxidative stress in the lungs of mice

**FIGURE 4:**
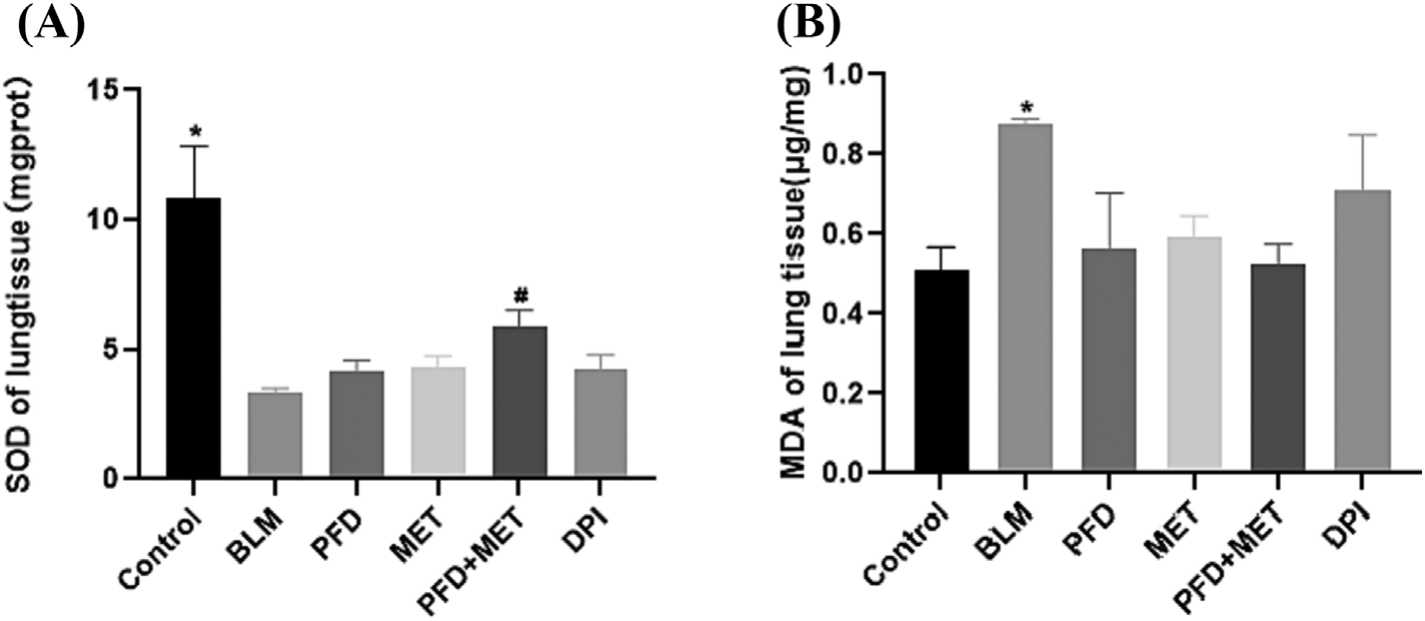
Superoxide dismutase (SOD) and malonaldehyde (MDA) level determination (A) compared with the control group, *p < 0.001. Compared with the bleomycin (BLM) group, #*p* < 0.05. Pirfenidone plus metformin can increase SOD levels in bleomycin‐induced pulmonary fibrosis in mice. (B) Compared with the BLM group, *p < 0.05. Compared to pirfenidone alone, pirfenidone plus metformin could reduce MDA levels in mice. Statistical analyses were performed by one‐way ANOVA with Tukey's test for comparisons between groups.

After BLM administration, SOD levels in the lungs of mice were significantly lower than those in the control group (*p* < 0.05). However, SOD levels were increased after treatment with pirfenidone or metformin. After the combined intervention, SOD levels in the lungs were similar to those in the control group. Compared with the BLM group, the difference was statistically significant (*p* < 0.05). These results suggested that the combination of the two treatments could increase the antioxidant level and reduce pulmonary peroxidation in mice. To further test whether metformin could reduce peroxide levels, we analysed MDA levels in fresh lung tissue. The results showed that BLM could increase MDA production in mice. Compared to pirfenidone alone, pirfenidone plus metformin could reduce MDA levels in mice. The combination of drugs enhanced the drug effects (Figure 4). These results suggest that metformin plays an antioxidative role by enhancing antioxidant capacity and reducing the production of peroxide. There was a stronger effect when metformin was combined with pirfenidone.

## 4 DISCUSSION

IPF is a type of fibrotic interstitial pneumonia. The aetiology of IPF is unknown, and the main clinical feature is progressive deterioration of lung function. The early symptoms of chest tightness and shortness of breath are easily ignored by patients. The median survival time after the diagnosis of IPF is only 2–3 years. This disease can be secondary to pulmonary hypertension, right heart failure, and deep vein thrombosis and can even increase the risk of lung cancer. IPF seriously affects the quality of life of patients and increases the economic burden on patients. At present, disease progression cannot be completely reversed unless lung transplantation is performed. The pathogenesis is known to involve inflammatory damage and oxidative stress. In 1987, Cartin found that the level of myeloperoxidase (MPO) was increased in the alveolar lavage fluid (BALF) of IPF patients.^6^ It was found that the ability alveolar inflammatory cells to release hydrogen peroxide (H_2_O_2_) was enhanced in IPF patients, and lipid hydroperoxide levels in the lungs were increased. Compared with that in normal individuals, the apoptosis rate of lung epithelial cells was increased, and the systemic oxidative stress level was positively correlated with the degree of pulmonary fibrosis.^7^ It has been proposed that the core pathway mediating fibrosis should be targeted to better antifibrotic drugs.^8^ Targeting age‐related oxidation/antioxidant imbalances has been identified as a core strategy.^9^ Antifibrotic drugs that block the source of reactive oxygen species (ROS) have effective antifibrotic effects.

Pirfenidone is an anti‐TGF‐β pyridone complex. It is a broad‐spectrum antifibrotic. It has anti‐inflammatory, antioxidant, and antifibrotic effects. It can inhibit pro‐inflammatory cytokines and promote fibrosis. It can clear ROS and inhibit lipid peroxidation. Pirfenidone improves symptoms such as dyspnoea in patients, but it is expensive and has limited efficacy. In addition, there are gastrointestinal and skin‐related adverse reactions.^10^ In the ASCEND trial, 16.5% of patients showed a 10% decrease in FVC% after 52 weeks of treatment with pirfenidone. The mortality rate increased gradually between 6 and 12 months. Pirfenidone can only moderately slow the decline in pulmonary function in IPF patients and cannot cure IPF. Treatment is accompanied by gastrointestinal side effects, light reaction skin disease, and other adverse reactions. A study evaluating two clinical trials that used pirfenidone showed that nearly all patients (765, 779 in total; 98%) reported at least one emergency adverse event.^10^ The adverse reactions were mainly gastrointestinal manifestations, skin diseases, and dizziness.^10^ Pirfenidone only moderately slows disease progression. The development of clinical drugs to treat pulmonary fibrosis has made little progress.

In this study, metformin enhanced the therapeutic effect of pirfenidone on mice with BLM‐induced pulmonary fibrosis. This study was conducted to investigate whether additional metformin therapy could improve the antifibrotic effect of pirfenidone. In this study, the lung tissue of BLM‐induced mice was subjected to H&E and Masson's trichrome staining, and the results showed the destruction of normal lung structure and obvious thickening of the alveolar septum. A large number of collagen fibres were deposited, and obvious pulmonary fibrosis was observed. Pirfenidone plus metformin protected against BLM‐induced pulmonary fibrosis in mice, reducing the Ashcroft score and decreasing HPO levels in lung tissue of BLM‐induced mice. The degree of pulmonary fibrosis in mice treated with pirfenidone plus metformin was reduced compared to that in mice treated with pirfenidone or metformin alone. HPO is a unique amino acid in collagen. The pathological mechanism of pulmonary fibrosis involves the excessive deposition of interstitial collagen in the lung, which affects the alveolar structure. Therefore, measuring HPO levels can reflect the levels of collagen, which indirectly represents the severity of pulmonary fibrosis. In this study, the Ashcroft score and HPO levels in the lung tissue of the six groups of mice were analysed. There was a significant correlation between the HPO level and the Ashcroft score.

An imbalance in oxidation–antioxidation is one of the key aetiological factors that promotes the occurrence and progression of pulmonary fibrosis. There is continuous production of ROS and the inability of antioxidant enzymes or other enzyme families to remove superoxides from the body in a timely manner. This accelerates the damage to cells. Among them, SOD is a key antioxidant enzyme. It can catalyse O_2_^−^ disproportionation to form oxygen and hydrogen peroxide. Hydrogen peroxide is broken down into water and O_2_ by catalase. SOD plays a key role in the oxidation/antioxidant balance of the body. MDA is a key oxidation product associated with lipid formation in O_2_^−^oxidized biofilms. MDA can cause the cross‐linking and polymerization of proteins, nucleic acids, and other macromolecules, leading to damage and cytotoxicity. The effects of ROS can be amplified through a chain reaction. MDA levels can reflect the degree of endogenous peroxidation in the body, and SOD levels can represent the antioxidant capacity of the body.

After BLM induction, SOD levels in the lungs of mice were significantly lower those that in the control group (*p* < 0.05). However, SOD levels were increased after treatment with pirfenidone or metformin. After the combined treatment, SOD levels in the lungs of mice were similar to those in the control group. Compared with the BLM group, there was a significant difference between the two groups (*p* < 0.05). It is suggested that pirfenidone plus metformin can reverse BLM‐induced peroxidation in mouse lungs by upregulating antioxidant levels. To further examine whether metformin could reduce peroxidation levels in the body, MDA levels were measured in fresh lung tissue. The results showed that BLM increased MDA production in mice. Metformin and pirfenidone plus metformin can reduce MDA levels (*p* < 0.05), showing a strong combination drug effect. These results suggest that metformin inhibits oxidative stress by enhancing antioxidant capacity and reducing peroxide production, and the combination of metformin plus pirfenidone has a stronger effect.

Oxidative stress is a state caused by an imbalance between the oxidation and antioxidant systems. When the overproduction of ROS exceeds the capacity of the antioxidant system or there is a reduction in antioxidants, it can lead to an imbalance in oxidation/antioxidation and then cause oxidative stress.^11^ Oxidative stress is an important cause of pulmonary fibrosis. During the progression of IPF, ROS are mainly derived from mitochondria and NOX4.^12^, ^13^ NOX4 is highly expressed in type II alveolar epithelial cells (AT2) in mouse models of pulmonary fibrosis and in patients with IPF. NOX4 can also produce many ROS.^13^ Oxidative stress can regulate cell growth, proliferation, and death by stimulating related genes and promoting the release of a variety of inflammatory mediators and cytokines, including the important transforming growth factor‐β (TGF‐β), thus promoting the development of pulmonary fibrosis. As the most commonly used hypoglycaemic drug, metformin can reduce the inflammatory response and oxidative stress independent of its hypoglycaemic effect.^2^, ^3^ Metformin can inhibit NOX4 and p38MAPK to reduce oxidative stress and inflammation and play a protective role.^14^, ^15^ To study the correlation between metformin and NOX4, NOX4 inhibitors were used to treat the model animals. In this study, the NOX4 inhibitor DPI alleviated BLM‐induced pulmonary fibrosis in mice. DPI could reduce pulmonary fibrosis and early alveolar inflammation, reduce lung collagen protein concentrations, and reduce HPO levels in the lung. These results indicate that the inhibition of NOX4 expression has potential application value in the treatment of pulmonary fibrosis.

This study demonstrated that metformin enhanced the antifibrotic effects of pirfenidone in a BLM‐induced pulmonary fibrosis mouse model. This enhancement may be related to antioxidative stress mechanisms and the inhibition of NOX4. This study laid the experimental foundation to further explore whether metformin can improve the antifibrotic effect of pirfenidone on IPF patients.

## AUTHOR CONTRIBUTIONS

Nana Liu and Hui Ma performed research, collected data and wrote the manuscript. Yanqiu Song performed statistical analysis. Ting Liu collected data. Hongyu Wang analysed and interpreted data. Naihao Yu designed research and performed research. All authors read and approved the final manuscript.

## CONFLICT OF INTEREST STATEMENT

The authors have no conflicts of interest to declare that are relevant to the content of this article.

## ETHICAL STATEMENT

Animal studies were approved by the Ethics Committee of Chest Hospital, Tianjin University and in compliance with the Guide for the Care and Use of Laboratory Animals.

## Data Availability

The datasets used and/or analysed during the current study are available from the corresponding author on reasonable request.
